# Supplementary material for: Marker-Assisted Recurrent Selection for Pyramiding Leaf Rust and Coffee Berry Disease Resistance Alleles in Coffea arabica L
Source: Genes (Basel). 2023 Jan 10;14(1):189. doi: 10.3390/genes14010189 (PMC9858729; doi:10.3390/genes14010189)
Supplement: Supplementary file 1 [file genes-14-00189-s001.zip › Supplement Table S1.pdf]

Marker-Assisted Recurrent Selection Applied for Pyramiding Leaf Rust and Coffee Berry Disease Resistance Alleles in *Coffea arabica* L.

Laura Maritza Saavedra[1], Eveline Teixeira Caixeta[1,2,*], Geleta Dugassa Barka[3], Aluízio Borém[4], Laércio Zambolim[1], Moysés Nascimento[5], Cosme Damião Cruz[6], Antonio Carlos Baião de Oliveira[2,7] and Antonio Alves Pereira[7]

[1]Instituto de Biotecnologia Aplicada à Agropecuária – Bioagro, Universidade Federal de Viçosa, Viçosa, Brazil
[2]Brazilian Agricultural Research Corporation (Embrapa), Embrapa Coffee, Brasília, Brazil
[3]Department of Applied Biology, School of Applied Natural Science, Adama Science and Technology University, Adama, Ethiopia
[4]Departamento de Agronomia, Universidade Federal de Viçosa, Viçosa, Brazil
[5]Departamento de Estatística, Universidade Federal de Viçosa, Viçosa, Brazil
[6]Departamento de Biologia Geral, Universidade Federal de Viçosa, Viçosa, Brazil
[7]Empresa de Pesquisa Agropecuária de Minas Gerais - Epamig, Viçosa, Brazil

[*]Corresponding author: eveline.caixeta@embrapa.br; ORCID 0000-0001-8850-6273

Table S1. Description of the SSR primers used in cross certification and diversity study of *Coffea arabica.*

Primer[a]	Sequences of Primers	Tm (°C)	Size (bp)
CaEST-002	F: GAAGGGACAAAGACGCCTAA	57.3	184
	R: CGACAGATGCAGGAATAAACTG		
CaEST-006	F: CAGAATTGTTGTGGAGGGAAC	57.9	227
	R: CGACAGATGCAGGAATAAACTG		
CaEST- 029	F: AGGAGATGCCTGTGACGAAC	53.7	199
	R: GGACGGAAAGATTCTGGCTTT		
CaEST- 030	F: CCCATGAAGACTTGCCAATA	54.2	171
	R: GGGAAATACAAGTGTTGCTG		
CaEST- 045	F: GCATCCTACCGAGTACATACAA	52.9	259
	R: TCCATCAACAACAACCGAAG		
CaEST- 048	F: TGAGACAAGCTATGGAGGAGGA	54.7	151
	R: AACCAGATCAACAGGGTAGGG		
CaEST- 071	F: ATGGAGAGGAAGACGCAACA	51.7	155
	R: CCTTATTGAAGACGCCCAAA		
CaEST- 072	F: TTGCTTGCTCCGCATCCTAC	53.7	197
	R: ATCGCTTCCAAGAGGCTTTC		
CaEST- 089	F: GTGAACCTCCCTTTCCCTTG	59.4	152
	R: ACTGGTCTCTCGTCTGTGAA		
CaEST- 102	F: GCTTCCTTACTTCCTTCCCTGA	60.3	208
	R: GGTTGCGTCAAACAAGTCAA		
CaEST- 088	F: CGCGTGGGAGATATTGAAGT	51.7	226
	R: AAGCGGCAGAAATCAGTGG		

[a] Ferrão et al. (2015); Melting temperature (Tm) and size of fragments generated in base pairs (bp).
