# Supplementary material for: Marker-Assisted Recurrent Selection for Pyramiding Leaf Rust and Coffee Berry Disease Resistance Alleles in Coffea arabica L
Source: Genes (Basel). 2023 Jan 10;14(1):189. doi: 10.3390/genes14010189 (PMC9858729; doi:10.3390/genes14010189)
Supplement: Supplementary file 1 [file genes-14-00189-s001.zip › Supplement Table S2.pdf]

Marker-Assisted Recurrent Selection Applied for Pyramiding Leaf Rust and Coffee Berry Disease Resistance Alleles in *Coffea arabica* L.

Laura Maritza Saavedra[1], Eveline Teixeira Caixeta[1,2,*], Geleta Dugassa Barka[3], Aluízio Borém[4], Laércio Zambolim[1], Moysés Nascimento[5], Cosme Damião Cruz[6], Antonio Carlos Baião de Oliveira[2,7] and Antonio Alves Pereira[7]

[1]Instituto de Biotecnologia Aplicada à Agropecuária – Bioagro, Universidade Federal de Viçosa, Viçosa, Brazil
[2]Brazilian Agricultural Research Corporation (Embrapa), Embrapa Coffee, Brasília, Brazil
[3]Department of Applied Biology, School of Applied Natural Science, Adama Science and Technology University, Adama, Ethiopia
[4]Departamento de Agronomia, Universidade Federal de Viçosa, Viçosa, Brazil
[5]Departamento de Estatística, Universidade Federal de Viçosa, Viçosa, Brazil
[6]Departamento de Biologia Geral, Universidade Federal de Viçosa, Viçosa, Brazil
[7]Empresa de Pesquisa Agropecuária de Minas Gerais - Epamig, Viçosa, Brazil

[*]Corresponding author: eveline.caixeta@embrapa.br; ORCID 0000-0001-8850-6273

Table S2. Molecular markers linked to coffee genes conferring resistance to *Hemileia vastatrix* and *Colletotrichum kahawae.*

Gene	Marker	Primer sequence	T (ºC)	Gene Distance (cM)
S*H*3: resistance to *H. vastatrix* [a]	*SP-M16-S*H*3	R: ATCTAGCTTTGGAACATCGT<br>F: TTAACTGGAAACTTGGCTTG	49	1.8
	*BA-124-12KF	R: TGCAGATTGATGGCACGTTA<br>F: TGATTTCGCTTGTTGTCGAG	56	0
	*BA-48-21O-f	R: ACTTGGCAGGCGTAATTGAA<br>F: ACAGTGAATTCCCCAAGCAC	52	0.6
	*Sat244	R:GCATACTAAGGAATTATCTGACTGCT<br>F: GCATGTGCTTTTTGATGTCGT	52	0
QTL: resistance to races I, II and pathotype 001 of *H. vastatrix* [b]	SSR 016	R: CCACACAACTCTCCTCATTC<br>F: ACCCGAAAGAAAGAACCAAG	65	3.7
	CaRHv8	R: TCTTAGCGCCATGAATAGCCA<br>F: ACCTTCTAGTGTTACCGAGGA	65	3
	CaRHv9	R: GTCTAAGACCAGAATCAGATGG<br>F: TGATGAAGAAGAGCGCATAGC	65	2.3
	CaRHv10_CAP	R: CAGCTGAACAACCGAACTCA<br>F: GACACATCGTGAACGTGGAG	65	22.4
C*k*-1: resistance to *C. kahawae* [c]	CBD-Sat235	R: GCAAATCATGAAAATAGTTGGTG<br>F: TCGTTCTGTCATTAAATCGTCAA	50	0
	CBD-Sat207	R: CAATCTCTTTCCGATGCTCT<br>F: GAAGCCGTTTCAAGCC	50	17.2

[a]Mahé et al. (2008), [b]Almeida et al. (2021), [c]Gichuru et al. (2008); Sequences, annealing temperature T (° C) and the distance between the gene and the marker

*Primer used in the analysis of genetic diversity
