# Supplementary material for: Marker-Assisted Recurrent Selection for Pyramiding Leaf Rust and Coffee Berry Disease Resistance Alleles in Coffea arabica L
Source: Genes (Basel). 2023 Jan 10;14(1):189. doi: 10.3390/genes14010189 (PMC9858729; doi:10.3390/genes14010189)
Supplement: Supplementary file 1 [file genes-14-00189-s001.zip › Supplement Table S4.pdf]

Marker-Assisted Recurrent Selection Applied for Pyramiding Leaf Rust and Coffee Berry Disease Resistance Alleles in *Coffea arabica* L.

Laura Maritza Saavedra[1], Eveline Teixeira Caixeta[1,2,*], Geleta Dugassa Barka[3], Aluízio Borém[4], Laércio Zambolim[1], Moysés Nascimento[5], Cosme Damião Cruz[6], Antonio Carlos Baião de Oliveira[2,7] and Antonio Alves Pereira[7]

[1]Instituto de Biotecnologia Aplicada à Agropecuária – Bioagro, Universidade Federal de Viçosa, Viçosa, Brazil
[2]Brazilian Agricultural Research Corporation (Embrapa), Embrapa Coffee, Brasília, Brazil
[3]Department of Applied Biology, School of Applied Natural Science, Adama Science and Technology University, Adama, Ethiopia
[4]Departamento de Agronomia, Universidade Federal de Viçosa, Viçosa, Brazil
[5]Departamento de Estatística, Universidade Federal de Viçosa, Viçosa, Brazil
[6]Departamento de Biologia Geral, Universidade Federal de Viçosa, Viçosa, Brazil
[7]Empresa de Pesquisa Agropecuária de Minas Gerais - Epamig, Viçosa, Brazil

[*]Corresponding author: eveline.caixeta@embrapa.br; ORCID 0000-0001-8850-6273

Table S4. Mean-rank and selection gains (%) of coffee hybrids obtained from the traits vegetative vigor (VIG), ripening fruit size (RFS), Cercosporiosis incidence (CER).

Hybrid Code	VIG	RFS	CER	Mean-rank	Gain %	Hybrid Code	VIG	RFS	CER	Mean-rank	Gain %
C4-10	2	8	3	4,3	92,8	C5-7	48	38	40	42,0	30,6
C3-9	3	17	9	9,7	84,0	C10-2	50	12	66	42,7	29,5
C2-12	1	24	4	9,7	84,0	C12-8	45	64	20	43,0	28,9
C3-8	5	5	25	11,7	80,7	C1-5	12	90	29	43,7	27,8
C9-4	7	16	21	14,7	75,8	C10-7	32	57	42	43,7	27,8
C3-12	28	1	26	18,3	69,7	C7-8	29	50	53	44,0	27,3
C8-5	54	4	10	22,7	62,5	C3-1	43	6	85	44,7	26,2
C12-1	34	29	16	26,3	56,5	C8-3	35	71	28	44,7	26,2
C10-10	11	29	39	26,3	56,5	C7-5	44	65	25	44,7	26,2
C12-6	47	33	6	28,7	52,6	C3-7	73	27	35	45,0	25,6
C9-6	13	70	5	29,3	51,5	C12-2	30	60	48	46,0	24,0
C4-6	25	46	18	29,7	51,0	C2-3	38	84	17	46,3	23,4
C2-10	6	49	36	30,3	49,9	C7-9	17	45	79	47,0	22,3
C8-7	12	49	31	30,7	49,3	C5-11	64	54	23	47,0	22,3
C10-9	14	56	22	30,7	49,3	C4-8	76	39	29	48,0	20,7
C11-7	63	7	24	31,3	48,2	C3-11	16	73	55	48,0	20,7
C5-8	22	32	42	32,0	47,1	C9-7	70	28	47	48,3	20,1
C7-3	51	23	24	32,7	46,0	C10-11	69	42	34	48,3	20,1
C6-11	60	20	18	32,7	46,0	C1-1	33	75	39	49,0	19,0
C12-9	9	78	15	34,0	43,8	C11-12	42	48	60	50,0	17,4
C7-2	26	76	2	34,7	42,7	C11-6	56	61	35	50,7	16,3
C8-2	4	30	70	34,7	42,7	C11-10	37	87	30	51,3	15,2

C3-10	53	14	38	35,0	42,1	C2-6	59	47	50	52,0	14,0
C4-9	8	34	64	35,3	41,6	C5-4	79	44	33	52,0	14,0
C9-5	31	32	44	35,7	41,0	C4-5	52	26	79	52,3	13,5
C2-5	68	21	21	36,7	39,4	C4-11	40	92	27	53,0	12,4
C4-7	10	33	67	36,7	39,4	C7-6	81	25	54	53,3	11,8
C5-5	19	36	57	37,3	38,3	C10-12	116	44	1	53,7	11,3
C1-2	96	9	8	37,7	37,7	C11-8	71	72	19	54,0	10,7
C3-5	39	31	43	37,7	37,7	C4-3	93	46	25	54,7	9,6
C5-2	36	40	38	38,0	37,2	C1-4	89	10	65	54,7	9,6
C5-9	24	55	35	38,0	37,2	C10-5	87	60	17	54,7	9,6
C8-9	27	35	54	38,7	36,1	C9-9	101	11	52	54,7	9,6
C1-11	80	37	7	41,3	31,7	C6-4	83	3	80	55,3	8,5
C2-9	18	94	13	41,7	31,1	C11-4	88	2	76	55,3	8,5
C9-10	21	64	40	41,7	31,1	C2-2	91	15	61	55,7	8,0
C6-7	20	85	67	57,3	5,2	C11-9	74	86	37	65,7	-8,5
C8-11	15	77	80	57,3	5,2	C7-11	115	19	63	65,7	-8,5
C2-11	118	19	37	58,0	4,1	C6-5	61	81	56	66,0	-9,1
C10-6	57	67	51	58,3	3,6	C8-4	94	59	45	66,0	-9,1
C1-9	90	18	67	58,3	3,6	C2-1	67	74	58	66,3	-9,6
C4-2	105	33	39	59,0	2,5	C10-8	72	53	74	66,3	-9,6
C7-12	66	64	48	59,3	1,9	C11-2	106	88	12	68,7	-13,5
C12-4	65	46	68	59,7	1,4	C8-10	111	22	73	68,7	-13,5
C8-8	92	64	23	59,7	1,4	C5-3	75	63	69	69,0	-14,0
C7-4	46	72	63	60,3	0,3	C5-12	86	89	32	69,0	-14,0
C12-5	62	73	46	60,3	0,3	C8-1	95	80	40	71,7	-18,5

C2-8	110	60	11	60,3	0,3	C9-12	112	62	41	71,7	-18,5
C5-1	78	43	61	60,7	-0,3	C11-11	103	52	60	71,7	-18,5
C11-1	77	57	48	60,7	-0,3	C9-2	49	82	85	72,0	-19,0
C9-8	108	47	28	61,0	-0,8	C11-3	85	68	63	72,0	-19,0
C6-3	100	24	60	61,3	-1,4	C1-10	97	62	59	72,7	-20,1
C12-3	117	58	14	63,0	-4,1	C6-8	113	37	72	74,0	-22,3
C12-10	83	59	49	63,7	-5,2	C10-1	99	69	78	82,0	-35,5
C6-6	23	95	75	64,3	-6,3	C4-4	109	59	86	84,7	-39,9
C1-12	58	51	84	64,3	-6,3	C9-1	102	91	62	85,0	-40,5
C5-10	84	41	68	64,3	-6,3	C2-7	98	70	87	85,0	-40,5
C7-1	78	45	71	64,7	-6,9	C7-7	107	66	83	85,3	-41,0
C12-7	104	13	77	64,7	-6,9	C6-10	82	93	82	85,7	-41,6
C4-1	41	73	81	65,0	-7,4	C9-11	114	79	70	87,7	-44,9
C10-3	55	83	58	65,3	-8,0	-	-	-	-	-	-
