# Supplementary material for: Marker-Assisted Recurrent Selection for Pyramiding Leaf Rust and Coffee Berry Disease Resistance Alleles in Coffea arabica L
Source: Genes (Basel). 2023 Jan 10;14(1):189. doi: 10.3390/genes14010189 (PMC9858729; doi:10.3390/genes14010189)
Supplement: Supplementary file 1 [file genes-14-00189-s001.zip › Supplement Table S6.pdf]

Marker-Assisted Recurrent Selection Applied for Pyramiding Leaf Rust and Coffee Berry Disease Resistance Alleles in *Coffea arabica* L.

Laura Maritza Saavedra[1], Eveline Teixeira Caixeta[1,2,*], Geleta Dugassa Barka[3], Aluízio Borém[4], Laércio Zambolim[1], Moysés Nascimento[5], Cosme Damião Cruz[6], Antonio Carlos Baião de Oliveira[2,7] and Antonio Alves Pereira[7]

[1]Instituto de Biotecnologia Aplicada à Agropecuária – Bioagro, Universidade Federal de Viçosa, Viçosa, Brazil
[2]Brazilian Agricultural Research Corporation (Embrapa), Embrapa Coffee, Brasília, Brazil
[3]Department of Applied Biology, School of Applied Natural Science, Adama Science and Technology University, Adama, Ethiopia
[4]Departamento de Agronomia, Universidade Federal de Viçosa, Viçosa, Brazil
[5]Departamento de Estatística, Universidade Federal de Viçosa, Viçosa, Brazil
[6]Departamento de Biologia Geral, Universidade Federal de Viçosa, Viçosa, Brazil
[7]Empresa de Pesquisa Agropecuária de Minas Gerais - Epamig, Viçosa, Brazil

[*]Corresponding author: eveline.caixeta@embrapa.br; ORCID 0000-0001-8850-6273

Table S6. Coffee hybrids associated with resistance to different races of *H. vastatrix* and that confers resistance to *C. kahawae*, identified by molecular markers.

a) Allele S_H3 (genotype Aa)									
C2-1	C2-5	C2-8	C2-11	C4-2	C4-5	C4-9	C12-1	C12-4	C12-7
C2-2	C2-6	C2-9	C2-12	C4-3	C4-6	C4-10	C12-2	C12-5	C12-8
C2-3	C2-7	C2-10	C4-1	C4-4	C4-8	C4-11	C12-3	C12-6	C12-9
									C12-10
b) QTL-GL2 (genotype BB)									
C1-1	C1-9	C6-4	C6-8	C8-2	C8-7	C8-11	C10-1	C10-9	C11-1
C1-2	C1-10	C6-5	C6-10	C8-3	C8-8	C9-4	C10-6	C10-10	C11-9
C1-4	C1-11	C6-6	C6-11	C8-4	C8-9	C9-5	C10-7	C10-11	C11-11
C1-5	C1-12	C6-7	C8-1	C8-5	C8-10	C9-10	C10-8	C10-12	-
c) QTL-GL2 (genotype Bb)									
C2-1	C2-9	C3-8	C4-3	C4-10	C5-7	C9-1	C9-12	C11-6	C12-7
C2-2	C2-10	C3-9	C4-4	C4-11	C5-8	C9-2	C10-2	C11-7	C12-8
C2-3	C2-11	C3-10	C4-5	C5-1	C5-9	C9-6	C10-3	C11-8	C12-9
C2-5	C2-12	C3-11	C4-6	C5-2	C5-10	C9-7	C10-5	C11-10	C12-10
C2-6	C3-1	C3-12	C4-7	C5-3	C5-11	C9-8	C11-2	C11-12	-
C2-7	C3-5	C4-1	C4-8	C5-4	C5-12	C9-9	C11-3	C12-1	-
C2-8	C3-7	C4-2	C4-9	C5-5	C6-3	C9-11	C11-4	C12-5	-
d) QTL-GL5 (genotype C_)									
C2-2	C3-1	C4-1	C4-7	C5-1	C5-8	C6-3	C6-8	C7-4	C7-9
C2-6	C3-8	C4-2	C4-8	C5-3	C5-9	C6-4	C6-10	C7-5	C7-11
C2-8	C3-9	C4-3	C4-9	C5-4	C5-10	C6-5	C6-11	C7-6	C7-12
C2-9	C3-10	C4-4	C4-10	C5-5	C5-11	C6-6	C7-1	C7-7	-

C2-10	C3-11	C4-5	C4-11	C5-7	C5-12	C6-7	C7-3	C7-8	-
e) Allele Ck-1 (genotype DD)									
C5-1	C5-2	C5-3	C5-5	C5-7	C5-4	C5-8	C5-9	C5-10	C5-11
f) Allele Ck-1 (genotype Dd)									
C1-2	C1-12	C3-8	C4-2	C4-9	C6-5	C7-1	C7-8	C10-5	C11-1
C1-4	C2-8	C3-9	C4-3	C4-10	C6-6	C7-2	C7-9	C10-7	C11-2
C1-5	C2-10	C3-10	C4-4	C4-12	C6-7	C7-3	C7-11	C10-8	C11-3
C1-9	C3-1	C3-11	C4-5	C5-12	C6-8	C7-4	C7-12	C10-9	C11-4
C1-10	C3-5	C3-12	C4-7	C6-3	C6-10	C7-6	C10-2	C10-10	C11-6
C1-11	C3-7	C4-1	C4-8	C6-4	C6-11	C7-7	C10-3	C10-11	C11-7

Coffee hybrids associated with resistance to different races of H. vastatrix: a) SH3 gene heterozygous (allele A); b) QTL of the linkage group 2 homozygous (allele B), c) QTL-GL2 heterozygous (genotype Bb); d) QTL of the linkage group 5 (allele C_) and Hybrids associated with resistance to C. kahawae e) Ck-1 gene homozygous (allele DD); f) Ck-1 gene heterozygous (allele Dd)
