# Supplementary material for: Marker-Assisted Recurrent Selection for Pyramiding Leaf Rust and Coffee Berry Disease Resistance Alleles in Coffea arabica L
Source: Genes (Basel). 2023 Jan 10;14(1):189. doi: 10.3390/genes14010189 (PMC9858729; doi:10.3390/genes14010189)
Supplement: Supplementary file 1 [file genes-14-00189-s001.zip › Supplement Table S7.pdf]

Marker-Assisted Recurrent Selection Applied for Pyramiding Leaf Rust and Coffee Berry Disease Resistance Alleles in *Coffea arabica* L.

Laura Maritza Saavedra[1], Eveline Teixeira Caixeta[1,2,*], Geleta Dugassa Barka[3], Aluízio Borém[4], Laércio Zambolim[1], Moysés Nascimento[5], Cosme Damião Cruz[6], Antonio Carlos Baião de Oliveira[2,7] and Antonio Alves Pereira[7]

[1]Instituto de Biotecnologia Aplicada à Agropecuária – Bioagro, Universidade Federal de Viçosa, Viçosa, Brazil
[2]Brazilian Agricultural Research Corporation (Embrapa), Embrapa Coffee, Brasília, Brazil
[3]Department of Applied Biology, School of Applied Natural Science, Adama Science and Technology University, Adama, Ethiopia
[4]Departamento de Agronomia, Universidade Federal de Viçosa, Viçosa, Brazil
[5]Departamento de Estatística, Universidade Federal de Viçosa, Viçosa, Brazil
[6]Departamento de Biologia Geral, Universidade Federal de Viçosa, Viçosa, Brazil
[7]Empresa de Pesquisa Agropecuária de Minas Gerais - Epamig, Viçosa, Brazil

[*]Corresponding author: eveline.caixeta@embrapa.br; ORCID 0000-0001-8850-6273

Table S7. Coffee hybrids genotypes, considering the presence of markers for the four loci of resistance to diseases in coffee.

Hybrid Code	Resistance genes				Hybrid Code	Resistance genes			
	S_H3	*LG2	**LG5	*Ck-1*		*S_H3*	*LG2	**LG5	*Ck-1*
C1-1	aa	BB	cc	dd	C7-5	aa	bb	C_	dd
C1-2	aa	BB	cc	dd	C7-6	aa	bb	C_	Dd
C1-4	aa	BB	cc	dd	C7-7	aa	bb	C_	Dd
C1-5	aa	BB	cc	dd	C7-8	aa	bb	C_	Dd
C1-9	aa	BB	cc	dd	C7-9	aa	bb	C_	Dd
C1-10	aa	BB	cc	dd	C7-11	aa	bb	C_	Dd
C1-11	aa	BB	cc	dd	C7-12	aa	bb	C_	Dd
C1-12	aa	BB	cc	dd	C8-1	aa	BB	cc	dd
C2-1	Aa	Bb	**cc**	**dd**	C8-2	aa	BB	cc	dd
C2-2	Aa	Bb	C_	dd	C8-3	aa	BB	cc	dd
C2-3	Aa	Bb	cc	dd	C8-4	aa	BB	cc	dd
C2-5	aa	Bb	cc	dd	C8-5	aa	BB	cc	dd
C2-6	Aa	Bb	C_	dd	C8-7	aa	BB	cc	dd
C2-7	Aa	Bb	cc	dd	C8-8	aa	BB	cc	dd
**C2-8**	**Aa**	**Bb**	**C_**	**Dd**	C8-9	aa	BB	cc	dd
C2-9	Aa	Bb	C_	dd	C8-10	aa	BB	cc	dd
**C2-10**	**Aa**	**Bb**	**C_**	**Dd**	C8-11	aa	BB	cc	dd
C2-11	Aa	Bb	cc	dd	C9-1	aa	Bb	cc	dd
C2-12	Aa	Bb	cc	dd	C9-2	aa	Bb	cc	dd
C3-1	aa	Bb	C_	Dd	C9-4	aa	BB	cc	dd
C3-5	aa	Bb	C_	Dd	C9-5	aa	BB	cc	dd

C3-7	aa	Bb	C_	Dd	C9-6	aa	Bb	cc	dd
C3-8	aa	Bb	C_	Dd	C9-7	aa	Bb	cc	dd
C3-9	aa	Bb	C_	Dd	C9-8	aa	Bb	cc	dd
C3-10	aa	Bb	C_	Dd	C9-9	aa	Bb	cc	dd
C3-11	aa	Bb	C_	Dd	C9-10	aa	BB	cc	dd
C3-12	aa	Bb	C_	Dd	C9-11	aa	Bb	cc	dd
**C4-1**	**Aa**	**Bb**	**C_**	**Dd**	C9-12	aa	Bb	cc	dd
**C4-2**	**Aa**	**Bb**	**C_**	**Dd**	C10-1	aa	BB	cc	dd
**C4-3**	**Aa**	**Bb**	**C_**	**Dd**	C10-2	aa	bb	cc	Dd
**C4-4**	**Aa**	**Bb**	**C_**	**Dd**	C10-3	aa	bb	cc	Dd
**C4-5**	**Aa**	**Bb**	**C_**	**Dd**	C10-5	aa	bb	cc	Dd
C4-6	Aa	Bb	cc	dd	C10-6	aa	BB	cc	dd
C4-7	Aa	Bb	C_	dd	C10-7	aa	BB	cc	Dd
**C4-8**	**Aa**	**Bb**	**C_**	**Dd**	C10-8	aa	BB	cc	Dd
**C4-9**	**Aa**	**Bb**	**C_**	**Dd**	C10-9	aa	BB	cc	Dd
**C4-10**	**Aa**	**Bb**	**C_**	**Dd**	C10-10	aa	BB	cc	Dd
**C4-11**	**Aa**	**Bb**	**C_**	**Dd**	C10-11	aa	BB	cc	Dd
C5-1	aa	Bb	C_	DD	C10-12	aa	BB	cc	dd
C5-2	aa	Bb	cc	DD	C11-1	aa	BB	cc	Dd
C5-3	aa	Bb	C_	DD	C11-2	aa	Bb	cc	Dd
C5-4	aa	Bb	C_	DD	C11-3	aa	Bb	cc	Dd
C5-5	aa	Bb	C_	DD	C11-4	aa	Bb	cc	Dd
C5-7	aa	Bb	C_	DD	C11-6	aa	Bb	cc	dd
C5-8	aa	Bb	C_	DD	C11-7	aa	Bb	cc	Dd
C5-9	aa	Bb	C_	DD	C11-8	aa	Bb	cc	dd

C5-10	aa	Bb	C_	DD	C11-9	aa	BB	cc	dd
C5-11	aa	Bb	C_	DD	C11-10	aa	Bb	cc	dd
C5-12	aa	Bb	C_	dd	C11-11	aa	BB	cc	dd
C6-3	aa	Bb	C_	Dd	C11-12	aa	Bb	cc	dd
C6-4	aa	BB	C_	Dd	C12-1	Aa	Bb	C_	dd
C6-5	aa	BB	C_	Dd	C12-2	Aa	bb	cc	dd
C6-6	aa	BB	C_	Dd	C12-3	Aa	bb	cc	dd
C6-7	aa	BB	C_	Dd	C12-4	Aa	bb	cc	dd
C6-8	aa	BB	C_	Dd	C12-5	Aa	Bb	cc	dd
C6-10	aa	BB	C_	Dd	C12-6	Aa	bb	cc	dd
C6-11	aa	BB	C_	Dd	C12-7	Aa	Bb	cc	dd
C7-1	aa	bb	C_	Dd	C12-8	Aa	Bb	cc	dd
C7-2	aa	bb	cc	Dd	C12-9	Aa	Bb	cc	dd
C7-3	aa	bb	C_	Dd	C12-10	Aa	Bb	cc	dd
C7-4	aa	bb	C_	Dd	-	-	-	-	-

*LG2 associated with the QTL of linkage group 2 and ** LG5 associated with the QTL of linkage group 5.
